# Benchmarking organic electrochemical transistors for plant electrophysiology

**DOI:** 10.3389/fpls.2022.916120

**Published:** 2022-07-22

**Authors:** Adam Armada-Moreira, Chiara Diacci, Abdul Manan Dar, Magnus Berggren, Daniel T. Simon, Eleni Stavrinidou

**Affiliations:** ^1^Laboratory of Organic Electronics, Department of Science and Technology, Linköping University, Norrköping, Sweden; ^2^Wallenberg Wood Science Center, Department of Science and Technology, Linköping University, Norrköping, Sweden; ^3^Umeå Plant Science Centre, Department of Forest Genetics and Plant Physiology, Swedish University of Agricultural Sciences, Umeå, Sweden

**Keywords:** plant electrophysiology, organic electrochemical transistor (OECT), organic electronics, Venus flytrap, *Arabidopsis thaliana*

## Abstract

Plants are able to sense and respond to a myriad of external stimuli, using different signal transduction pathways, including electrical signaling. The ability to monitor plant responses is essential not only for fundamental plant science, but also to gain knowledge on how to interface plants with technology. Still, the field of plant electrophysiology remains rather unexplored when compared to its animal counterpart. Indeed, most studies continue to rely on invasive techniques or on bulky inorganic electrodes that oftentimes are not ideal for stable integration with plant tissues. On the other hand, few studies have proposed novel approaches to monitor plant signals, based on non-invasive conformable electrodes or even organic transistors. Organic electrochemical transistors (OECTs) are particularly promising for electrophysiology as they are inherently amplification devices, they operate at low voltages, can be miniaturized, and be fabricated in flexible and conformable substrates. Thus, in this study, we characterize OECTs as viable tools to measure plant electrical signals, comparing them to the performance of the current standard, Ag/AgCl electrodes. For that, we focused on two widely studied plant signals: the Venus flytrap (VFT) action potentials elicited by mechanical stimulation of its sensitive trigger hairs, and the wound response of *Arabidopsis thaliana*. We found that OECTs are able to record these signals without distortion and with the same resolution as Ag/AgCl electrodes and that they offer a major advantage in terms of signal noise, which allow them to be used in field conditions. This work establishes these organic bioelectronic devices as non-invasive tools to monitor plant signaling that can provide insight into plant processes in their natural environment.

## Introduction

Plants are the most widespread organisms on the planet, representing the majority of Earth’s biomass (Bar-On et al., 2018). These organisms, the product of millions of years of evolution, are highly developed biosensors, capable of monitoring a myriad of external stimuli such as water availability, temperature, and soil composition, among many others (Yu et al., 2021). The stimuli sensed by plants are transduced *via* different signaling pathways, including hydraulic, chemical, and electrical signals (Choi et al., 2016). While the importance of electrical signals for plant signaling has been widely reported, it is a very complex phenomenon, as recently reviewed by Klejchova et al. (2021). Indeed, when considering biological signaling, electrical signals do not occur isolated. Instead, they are intrinsically related to ionic transients and plant hormonal responses (Farmer et al., 2020; Suda et al., 2020; Grenzi et al., 2021). Monitoring plant responses to various stimuli in high resolution will not only advance our knowledge on basic plant science, which can be used to improve plant acclimation to biotic and abiotic stress, but also provide a handle for interfacing plants with technology, aiding the development of advanced and green technology.

A notable example of plant electrical signaling is the Venus flytrap (VFT) action potential. These fast action potentials are one of the most well-known plant signals, having been first described in the 19th century (Burdon-Sanderson, 1873). These electrical signals, characterized by an “all-or-nothing” response, fast propagation, and constant amplitude (Gilroy and Trewavas, 2001), are elicited by the activation of mechanosensitive trigger cells and lead to the sudden closure of the trap, allowing for the digestion of small insects and arachnids (Hedrich and Neher, 2018).

In another relevant example, a different and slower type of plant signals is the slow wave potential. This signal, induced by external stressors, consists of a transient depolarization of irregular shape and duration (Vodeneev et al., 2015), and has been associated with the activation of cellular defense mechanisms, such as the synthesis of the defense-related hormone jasmonate (Mousavi et al., 2013; Nguyen et al., 2018) and ethylene (Marhavý et al., 2019). While these signals have an intracellular origin, it is possible to monitor them by changes in the leaf surface potential, which are called wound-activated surface potential changes (WASPs) (Mousavi et al., 2013).

Overall, even though this signaling mechanism is quite widespread and carries an enormous amount of information, the field of plant electrophysiology is still in its infancy when compared to its animal counterpart. Most common techniques in this field are restricted to cumbersome data acquisition setups and laboratory conditions, requiring the use of Faraday cages and physical immobilization of plants (Dufil et al., 2021). Other techniques, especially applied to intracellular recordings, are not suitable for monitoring environmental electrophysiological responses, since they wound the plant and consequentially alter their electrical behavior (Salvador-Recatalà et al., 2014). Thus, there is a need to develop new tools that allow *in situ* monitoring in a non-invasive manner.

Recent reviews in plant electrophysiology highlight the need for this field to be considered as an interdisciplinary challenge in order to attain significant knowledge (Li et al., 2021). Indeed, the development of devices and materials capable of interfacing with plants, for a myriad of functions, has been increasing, bringing together material science and plant biology (Lew et al., 2020; Dufil et al., 2021). A new contender to advance this effort is the field of organic electronics.

While the current standard for plant electrophysiology remains Ag/AgCl electrodes (Volkov et al., 2011, 2019), or other metal inorganic electrodes (Rhodes et al., 1996; Brette and Destexhe, 2012; Chatterjee et al., 2015, 2017; Ríos-Rojas et al., 2015; Kim et al., 2018; Chong et al., 2019), novel approaches from the realm of organic electronics are now been explored. These include self-adhering poly(3,4-ethylenedioxythiophene) polystyrene sulfonate (PEDOT:PSS)-based surface electrodes, which are flexible and can thus conform to plant anatomy (Meder et al., 2021), as well as the development of an ionic electrode capable of interfacing hairy plant tissues with metal electrodes that allows for a significant improvement in signal-to-noise ratio (SNR) and maintains signal stability regardless of plant movements (Luo et al., 2021).

Among organic electronic devices, the organic electrochemical transistor (OECT) represents an optimal platform for interfacing living systems (Nawaz et al., 2021) as it operates at low voltages and has an electrolyte as an integral part of the device. The OECT is a three terminal device where source and drain electrodes are connected by a thin layer of an organic (semi) conductor, while the gate electrode is separated by an electrolyte. Typically, in OECTs, organic mixed-ionic electronic conductors (OMIECs), such as the conjugated polymer PEDOT:PSS, are used as the channel material (Stavrinidou et al., 2013; Paulsen et al., 2020). Upon bias application between gate and source, ions are driven from the electrolyte into the channel resulting in doping or dedoping of the OMIEC layer and therefore changing the channel conductivity (Rivnay et al., 2018). The ions can penetrate throughout the OMIEC layer volume, giving rise to a volumetric capacitance, which allows high signal amplification, and thus operation in low voltage regime (Proctor et al., 2016; Volkov et al., 2017). Additionally, OECTs can be fabricated on ultra-thin, flexible and conformable substrates (Khodagholy et al., 2013; Cea et al., 2020). They are miniaturized devices with high amplification and ensure high SNR, essential characteristics for *in vivo* recording.

One of the first uses of OECTs as tools for electrophysiology was described by Malliaras and colleagues the authors developed OECT arrays to successfully measure epileptiform discharges in rat brain, demonstrating higher SNR compared to surface electrodes (Khodagholy et al., 2013). The application of OECTs as tools for mammalian electrophysiology has become increasingly widespread and validated, with several innovations in both surface and implantable technologies in the last years (Bai et al., 2019). On the other hand, reports on the use of OECTs for plant monitoring and electrophysiology are much sparser in literature. Two notable examples focus on the successful use of enzymatically functionalized OECTs for long-term monitoring of glucose and sucrose in trees (Diacci et al., 2021) and on a first demonstration of OECTs as tools for plant electrophysiology, where these devices were able to record the action potential of the carnivorous VFT, with increased SNR compared to Ag/AgCl electrodes (Bischak et al., 2020). However, this last example is only a demonstration that does not fully characterize these devices as electrophysiological tools.

**FIGURE 1:**
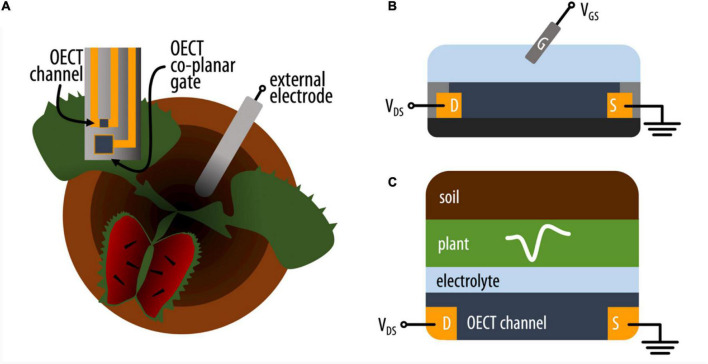
**(A)** Schematic representation of the experimental setup where an OECT (top view) with channel and co-planar gate electrode is attached on the lobe of the VFT and an external electrode is placed in the soil. **(B)** Schematic (side) representation of an OECT where the current in the conducting polymer channel between source (S) and drain (D) electrodes is modulated by a voltage applied between the electrolyte and channel *via* a gate electrode (G). **(C)** Simplified schematic of the various electrolytic components interfacing with the OECT channel on the VFT lobe (side view).

Thus, we aimed to benchmark OECTs for plant electrophysiology (Figure 1) using two well-known plant electrical signals as biological models: action potential of VFT and wound response of *Arabidopsis thaliana*. In this work, we compare OECTs to a PEDOT:PSS electrode (equivalent in size and composition) and to a Ag/AgCl electrode (the current gold standard for plant electrophysiology). We also compare the performance of the different devices in less controlled conditions, where a Faraday cage is not used to minimize external noise.

## Materials and methods

### Organic electrochemical transistor fabrication

A 125 μm-thick polyethylene naphthalate foil (PEN, Teonex Q65HA, Peutz Folien GmbH, Germany) was cut in a circular substrate with 10.2 cm diameter. The substrate was cleaned with water and acetone, then vacuum baked for 90 s at 120°C. Layers of 2 nm of chromium (Cr), for a better metal adhesion, and 50 nm gold (Au) were evaporated onto the clean surface. Photolithography (MA/BM6 Mask Aligner, SUSS MicroTec SE, Germany) and a Shipley 1805 positive resist were used to pattern the gold contacts, wiring, channel and gate/s. The substrate was then wet etched in I_2_/KI solution for Au, and with a chromium etcher for the chromium layer while the remaining resist was stripped with acetone. A thin film of PEDOT:PSS (Clevios PH1000) mixture with 5% (v/v) ethylene glycol (EG), 1% (v/v) (3-Glycidyloxypropyl)trimethoxysilane (GOPS), and dodecylbenzenesulfonic acid (1 drop per 5 ml) was deposited by spin-coating and patterned using a Shipley 1813 positive resist. The PEDOT:PSS layer was then dry etched with CF_4_/O_2_ reactive ions, in order to create channels and gates. The remaining resist was stripped again with acetone. In the end, the substrate was encapsulated, to ensure wire insulation with SU-8 2010 (MicroChem) and openings on the active areas were created by wet etching with developer mr-Dev 600 (Microresist Technology). Chemicals were used as received from Sigma-Aldrich unless stated otherwise.

### Plant material

Venus flytrap plants were acquired from Plantagen (Sweden) and kept in a greenhouse with controlled temperature and humidity (22/18°C light/dark, 12 h photoperiod, 60% relative humidity), and watered with DI water. *A. thaliana* were seeded and grown in a controlled growth chamber (IntellusUltra Connect, Percival Scientific, IA, United States), with a 12 h photoperiod, at 18°C and 80% relative humidity.

### Electrophysiological recording of Venus flytrap action potentials

Venus flytrap plants were removed from the greenhouse and left to acclimate to the experimental room for at least 10 min before experiments were performed. For Ag/AgCl and PEDOT:PSS electrode recordings, the electrode was placed on a trap, using Signa gel as an electrolyte, and a Ag/AgCl electrode in the soil was used as a reference electrode. One trigger hair was touched using a wooden stick after acquiring 20 s of baseline recording (no activity). Data was acquired using a InfiniiVision 3000A X-Series digital oscilloscope (Keysight Technologies, CA, United States). For OECT recordings, the source-drain channel of the OECT was placed on a trap, using Signa gel as an electrolyte, and a Ag/AgCl electrode in soil was used as the gate electrode. The device was biased with V_*DS*_ = –0.4 V and V_*GS*_ = 0.3 V. Data was recorded using a Keithley SourceMeter 2612B (Tektronix, OR, United States) and custom Labview software. For recordings inside the Faraday cage, an extra Ag/AgCl electrode was placed in soil and connected to the Faraday cage, in order to ground the whole system.

### Electrophysiological recording of *Arabidopsis thaliana* laser-induced wound response

Five-week-old *A. thaliana* plants were removed from the growth chamber and left to acclimate in the recording setup for c. 20 min. At this point, a Ag/AgCl electrode was placed in the damp soil and one of the recording devices was interfaced with one of the leaves, using a 10 mM KCl and 20 wt% PVA in DI water solution as an electrolyte. For Ag/AgCl and PEDOT:PSS electrode recordings, the Ag/AgCl electrode in soil was used as the reference electrode. For OECT recordings, this electrode was used as the gate electrode and the device was biased with V_*DS*_ = –0.4 V and V_*GS*_ = 0.3 V, which resulted in the highest signal amplification (Supplementary Figure 1). For recordings inside the Faraday cage, an additional Ag/AgCl electrode was place in the soil, and used to ground the whole system. A 7 W laser (450 nm, Sainsmart, KA, United States) was focused on the main vein 1 cm distally away from the recording device, in order to induce a wound with 1 mm diameter. Baseline activity was recorded for at least 20 s before the laser was triggered. The laser was active for 1 s at 50% laser power, which was sufficient to burn through the leaf. Wound response was recorded for at least 100 s following the laser action, using a Keithley SourceMeter 2612B for OECT or a ME2100-System (Multichannel Systems, Germany) for the Ag/AgCl or PEDOT:PSS electrodes.

### Data analysis

All experiments were performed with at least three different plants. A detailed characterization of the full datasets can be found in Supplementary Table 1. Data acquired from the digital oscilloscope and the ME2100-System were downsampled to 100 Hz to match that acquired by the Keithley SourceMeter. For VFT action potentials, data were filtered with an analog Bessel highpass filter (0.01 Hz, order 2) to remove baseline wandering. The different action potentials were aligned by their steepest deflection point (local minima of the first temporal derivative). If more than one action potential was recorded in one trap, these were averaged and considered to be *N* = 1. For *A. thaliana* data, the different WASPs were aligned by the timing of laser on. For parameter quantification, all waveforms were smoothed with a gaussian averaging filter, with window size of 200 ms and 500 ms, for VFT and *A. thaliana*, respectively. Data were analyzed with custom Matlab code and GraphPad Prism.

## Results

### Organic electrochemical transistor configuration for plant electrophysiology

**FIGURE 2:**
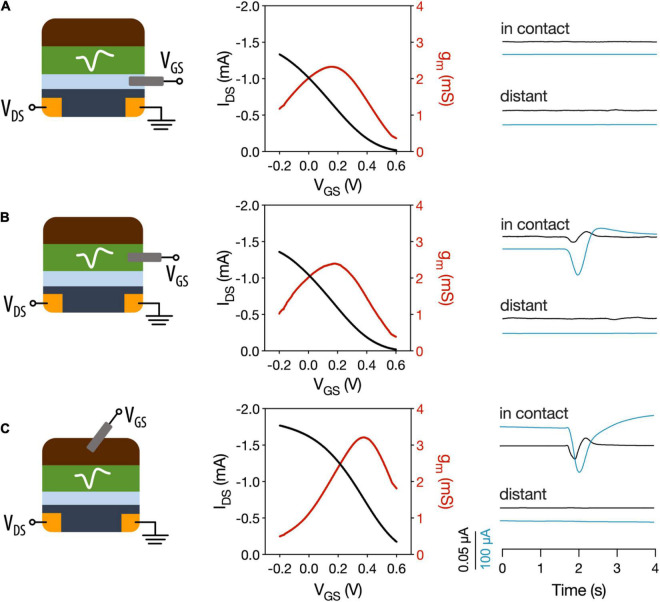
Different OECT configurations for plant electrophysiology. OECT transfer curves and transconductance and VFT action potentials recorded with **(A)** OECT co-planar PEDOT:PSS gate electrode. **(B)** Ag/AgCl electrode in a non-electrically active plant tissue as the gate electrode. **(C)** Ag/AgCl electrode in soil used as gate electrode. In contact refers to a recording of the trap where the OECT channel is attached. Distant corresponds to recordings in which a different trap was stimulated. Black trace: gate current. Blue trace: drain current.

The motivation of using OECTs as plant electrophysiological tools relies in the inherent amplification of the OECT device where small changes on the gate voltage are translated as larger changes in the OECT channel current. We hypothesize that the plant signals can act as voltage modulators at the gate electrode. In order to optimize the electrophysiology recordings with OECTs, different OECT configurations were tested using the VFT as the model system. In all configurations, the OECT channel was placed on a lobe of one of the traps, using the commercially available Signa gel as the interfacing electrolyte. However, we explored three different ways to interface the gate electrode: (i) a PEDOT:PSS electrode was placed on the same lobe with the OECT channel and was electrolytically connected to plant and channel (Figure 2A and Supplementary Figure 2 for alternative representation); (ii) a Ag/AgCl electrode was attached on a non-electrically active plant surface (Figure 2B); and (iii) a Ag/AgCl electrode was immersed in the soil (Figure 2C). In order to study the modulation of the OECT channel current *via* the gate electrode, we measured the transfer curve in the various configuration (V_*DS*_ = −0.4 V and V_*GS*_ from −0.2 to 0.6 V). The transfer curves showed that efficient modulation of the channel current occurs in all configurations with slightly improved modulation when the gate electrode is placed in the soil. Quantitatively, this is shown by the maximum transconductance value that corresponds to the derivative of the transfer curve and hence the change on the channel current due to a change on the gate potential.

Then, action potentials were recorded in the different configurations, by stimulating either the trap in which the OECT channel was attached or a distant trap. First, we observed that when a co-planar gate electrode is used, we were not able to record the action potential. In contrast when the gate electrode is placed on the plant or in the soil the action potential can be recorded as a change in the OECT channel current. These results show that, in order to efficiently record the plant signals, the plant must be part of the OECT circuit with gate voltage being applied between plant and OECT channel either *via* soil or a non-electrically active plant tissue. Furthermore, the action potential was recorded only when the OECT channel was attached on the stimulated trap and not at a distant one. This shows that the plant signal is local and does not travel across the whole plant, which is in line with previous reports (Volkov et al., 2007; Suda et al., 2020) that show that the signal does not travel beyond the petiole.

Thus, for the following experiments, a Ag/AgCl electrode placed in the soil was used as the gate electrode of the device, which is in line with previous studies of OECTs as electrophysiological tools in plants (Bischak et al., 2020) and in mammalian brain (Khodagholy et al., 2013).

### Fast plant signals: Venus flytrap action potentials

**FIGURE 3:**
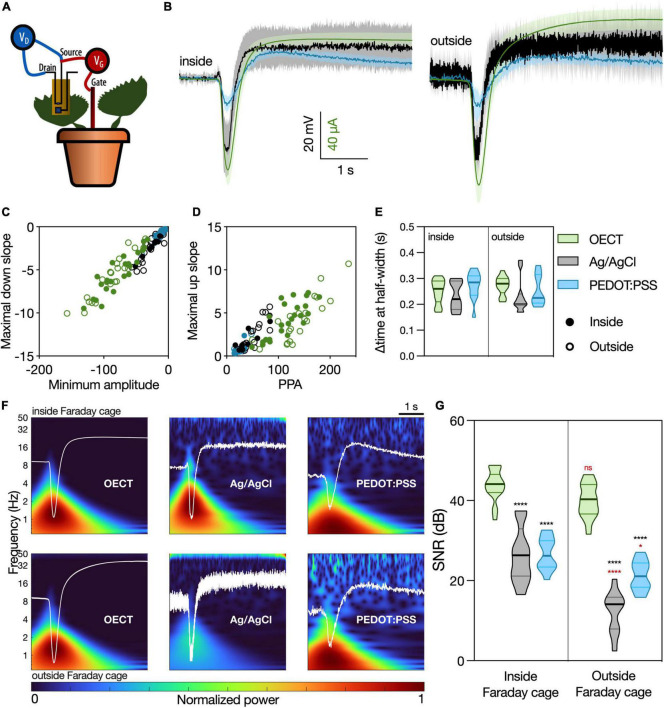
Venus flytrap (VFT) action potentials recorded with different devices. **(A)** Schematic representation of the recording setup. The OECT channel was attached on a lobe of a VFT trap, using a commercially available electrophysiology gel as an electrolyte. Gate voltage (V_*GS*_) was applied between source and a Ag/AgCl electrode in the soil. Drain voltage (V_*DS*_) was applied across the OECT channel. For recordings with Ag/AgCl or PEDOT:PSS electrodes, the recording electrodes were attached on the trap, and a Ag/AgCl electrode in soil was used as reference. For the recordings inside of the Faraday cage, an extra Ag/AgCl electrode was placed in the soil and used to ground the whole system. **(B)** Average waveform acquired with the different devices: OECT (green trace), Ag/AgCl electrode (black trace), and PEDOT:PSS electrode (blue trace). Data represented as mean ± 95% CI. *N* = 9–24 traps from 4 to 10 independent plants. Quantification of the **(C)** linear relationship between the maximal down slope and minimum amplitude; **(D)** linear relationship between the maximal up slope and the peak-to-peak amplitude of the action potential; **(E)** duration of action potential, characterized by the delta time at half-width. No significant differences were found in any of the considered parameters, using a simple linear regression and statistical comparison of slopes [for panels **(C,D)**] or a two-way ANOVA considering the parameters “device” and “Faraday cage” [for panel **(E)**]. **(F)** Time-frequency domain normalized magnitude scalograms of the average waveform acquired with the different devices, inside and outside of the Faraday cage. The average waveform is overlaid in white. **(G)** Quantification of SNR for the different waveforms, *N* = 9–20. *p < 0.05, ****p < 0.0001 using a two-way ANOVA considering the parameters “device” and “Faraday cage,” followed by Tukey’s multiple comparison test. In black, differences within the same “cage” condition; in red, differences in the same “device” condition. In **(E,G)**, data are represented in violin plots, where the width of the shaded area represents the proportion of data points at any given zone, with lines at median and quartiles.

Given their importance in the field of plant electrophysiology, the VFT action potentials were chosen to first investigate the use of OECTs as tools in plant electrophysiology. The OECT channel was placed on a trap, using the commercially available Signa gel as the interfacing electrolyte and a Ag/AgCl electrode in the soil as the gate electrode (Figure 3A) as described in the previous section. For recordings with Ag/AgCl and PEDOT:PSS electrodes, the recording electrode was placed on the trap and a Ag/AgCl electrode in soil as the reference electrode. The average waveforms recorded with the different devices (Figure 3B) show high correlation among themselves (Supplementary Table 2), which implies that the OECT performed similarly to the electrodes in recording this phenomenon. Importantly, three different OECTs were used to record these signals, to ensure proper reproducibility among devices. The waveforms obtained from the different OECTs were equivalent (Supplementary Figure 3).

Different waveform parameters were quantified in order to compare in detail the similarity of the different devices in resolving this signal (Supplementary Figure 4). The relationship between the peak signal amplitude and slope (Figure 3C), as well as the relationship between the peak-to-peak amplitude (PPA) and the slope between signal minimum and maximum (Figure 3D), showed similar behavior for all considered devices, both inside and outside of the Faraday cage (Supplementary Table 3). Furthermore, the time interval at half-width was considered to quantify the temporal component of the action potential (Figure 3E). Again, no differences were found between the different devices. Taking all these quantifications into account, we conclude that the OECT is as efficient in recording the VFT action potentials as the current standard for plant electrophysiology (Ag/AgCl electrode) and the similar in size and composition PEDOT:PSS electrode.

Signal-to-noise ratio of the different devices was then calculated as the peak amplitude of the waveform (current or voltage) divided by the standard deviation of the baseline recording (no stimulation), a widely used method of computing SNR found in literature (Khodagholy et al., 2013; Bischak et al., 2020). In Figure 3F, the time-frequency domain of the average waveforms, a visual representation of the signal power at each frequency band and timepoint, shows a much clearer signal resolution for OECT compared to other devices, especially outside of the Faraday cage. This finding is then quantified in Figure 3G, where the OECT shows a higher SNR compared to the other devices, either inside or outside of the Faraday cage. Additionally, the signal recorded with the OECT does not get disrupted outside of the Faraday cage, unlike those recorded with the Ag/AgCl and PEDOT:PSS electrodes.

### Slow plant signals: *Arabidopsis thaliana* wound-activated surface potential changes

As previously mentioned, slow wave potentials and, more specifically, wound-induced potential changes, are also relevant plant electrical signals. However, when studying wounding responses, it is very common to find signal artifacts that arise from the movement induced by the wounding stimulus and not the wounding itself (Degli Agosti, 2014; Luo et al., 2021). Thus, to avoid such artifacts, we chose to study a laser-induced wound response. Since WASP intensity weakens with increasing distance from site of wounding (Stahlberg et al., 2006), we chose to record 1 cm away, in the proximal direction, from the laser and keep that distance constant. Furthermore, it is also known that WASP amplitude depends on the intensity of the stimulus (Vodeneev et al., 2015), which led us to set the 7 W laser at a constant intensity of 50% of laser power for 1 s.

**FIGURE 4:**
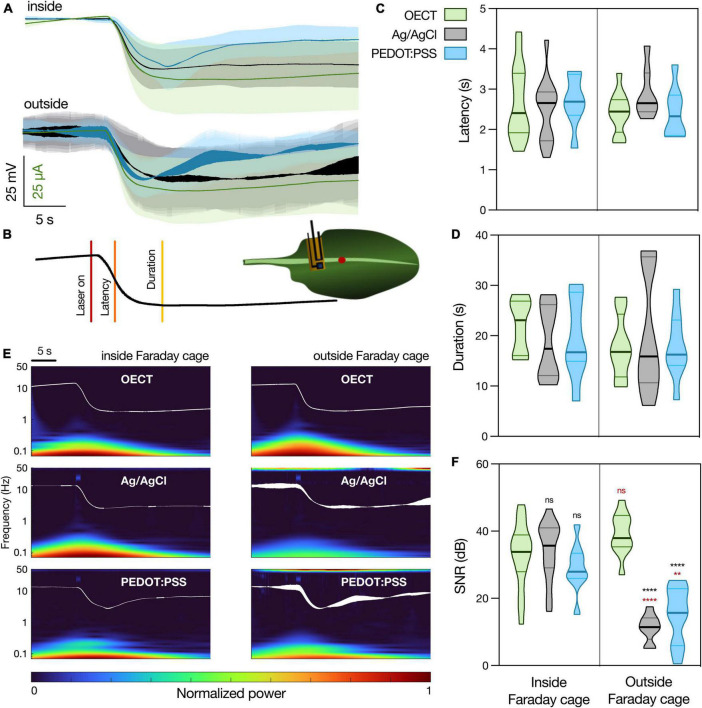
*Arabidopsis thaliana* WASP triggered by laser recorded with different devices. **(A)** Average waveform recorded with the different devices, inside and outside the Faraday cage: OECT (green trace), Ag/AgCl electrode (black trace), and PEDOT:PSS electrode (blue trace). Data represented as mean ± 95% CI. ***N*** = 10–20 leaves from 3 to 5 different plants. **(B)** Schematic representation of experimental setup. Laser was shined on a leaf for 1 s at 50% laser power and the different devices were in contact with the leaf less than 1 cm away, in a proximal direction, from the laser. The parameters considered for the characterization of the WASP were latency (time to reach half of maximal amplitude) and duration (time from laser on to recovery). **(C)** Quantification of latency and **(D)** duration of WASPs recorded with the different devices. No statistically significant differences were found. ***N*** = 7–18. **(E)** Time-frequency analysis of the average waveform acquired with the different devices, inside and outside of the Faraday cage. **(F)** Quantification of SNR for the different waveforms, ***N*** = 9–19. **p < 0.01, ****p < 0.0001 using a two-way ANOVA considering the parameters “device” and “Faraday cage,” followed by Tukey’s multiple comparison test. In black, differences within the same “cage” condition; in red, differences in the same “device” condition. In **(C,D,F)**, data are represented in violin plots, where the width of the shaded area represents the proportion of data points at any given zone, with lines at median and quartiles.

The different devices were then interfaced with an *A. thaliana* leaf, using a solution of 10 mM KCl and 20 wt% PVA in DI water as electrolyte. Similarly to the experimental setup for recording the VFT action potential, a Ag/AgCl electrode in the soil was used as the gate electrode for OECT recordings or as the reference electrode for PEDOT:PSS and Ag/AgCl electrode recordings. For recordings inside of the Faraday cage, an additional Ag/AgCl electrode was placed in the soil and used to ground the system. The average waveform recorded with the different devices (Figure 4A) is similar to those found in literature (Mousavi et al., 2013; Nguyen et al., 2018). Additionally, as previously described in literature, we did not find any spontaneous electrical activity unrelated to the wounding signal (Mousavi et al., 2013). All the obtained waveforms share a high correlation between the different devices and also between different Faraday cage conditions (Supplementary Table 2), implying that the OECT can resolve this biological signal as efficiently as the other electrodes. While the signals are similar, the WASPs present an increased variability in their response tail. This was already expected since WASP architecture is known to be quite variable, even within the same plant species (Mousavi et al., 2013; Farmer et al., 2020), which accounts for the increased error associated with the duration/recovery of the recorded WASPs.

In order to finely quantify the waveform similarity between the different devices, two different WASP parameters were considered, as depicted in Figure 4B. These were latency (Figure 4C, the time interval between wounding and reaching half peak amplitude) and duration (Figure 4D, the time interval between wounding and recovery, measured as the zero in the signal’s first temporal derivative). Using a two-way ANOVA considering the parameters “device” and “Faraday cage,” no statistically significant differences were found between the different samples. Considering the waveform and parameter similarity, it is possible to conclude that the OECT is able to record this biological signal in a manner comparable to the current gold standard.

Finally, the frequency content of the different waveforms was investigated (Figure 4E) and a SNR analysis was performed (Figure 4F). When the recordings were performed inside of the Faraday cage, all the devices show a similar time-frequency spectrum, although it is possible to observe some noise at around 40 Hz caused by the laser in the spectrums of the PEDOT:PSS and Ag/AgCl electrodes. This is then translated into similar values of SNR between the different devices. However, when the recordings were performed outside of the Faraday cage, it is possible to observe an increase in noise levels (50 Hz) in both PEDOT:PSS and Ag/AgCl electrode recordings. Similarly, this is translated into a significant decrease in the SNR for these devices, while the OECT maintains a high SNR, which highlights an advantage of using this latter device for plant electrophysiology, especially outside of laboratory conditions.

## Discussion

In this work, we set out to benchmark OECTs for plant electrophysiology, comparing their performance to that of PEDOT:PSS and Ag/AgCl electrodes. We were able to conclude that OECTs can resolve the biological signals as efficiently as the tested electrodes and that these organic electronic devices offer a significant improvement in the SNR of the recordings, especially when moving away from laboratory conditions. This is, to our knowledge, the first report that thoroughly characterizes these devices for plant electrophysiology, in a controlled and quantified manner, considering two different types of electrical signals. Importantly, while surface electrical recordings do not allow for the determination of the cellular origin of the signals, this approach represents a compromise between spatial resolution and invasiveness and remains a relevant method to extract meaningful information about plant electrophysiology.

Regarding the VFT action potential, while the waveforms attained are similar to some found in literature (Bischak et al., 2020), there is a large variability in action potential shape reported over the years (de Bakker et al., 2021; Meder et al., 2021). While a more comprehensive study on the origin of this variability remains to be conducted, special attention should be paid to the experimental setup and data filtering in plant electrophysiology. Indeed, since electrophysiology has been developed for mammalian systems, most commercially available electrophysiology systems are not suitable for plant signal recording. For this application, it is required that the employed system has the capacity to record high amplitude signals and has no hardware highpass filter (such as the commonly used 0.1 Hz highpass filter). Along the same line, data filtering can deeply impact the recorded signal. For example, for neuronal signals, it is possible to bandpass them to extract different components only because a great deal of research was devoted to isolate and quantify the different frequency contents of those signals. Such knowledge does not yet exist for plant electrical signals. Thus, even seemingly innocuous data treatment can mask some aspects of these signals. In practical terms, while data acquisition and treatment in plant electrophysiology still needs to be further elucidated and optimized, this discussion brings to light the need to create field-wide standards and analyses.

Regarding the values found for SNR, our study is in line with previous reports that state that VFT action potentials recorded with OECTs have a SNR of 1250, compared to that of Ag/AgCl of 11 (Bischak et al., 2020), which corresponds to a difference of around 40 dB between the devices. While these findings are in line with our results, this study does not mention if the recordings were performed inside a Faraday cage nor does it mention if data filtering was performed.

Regarding *A. thaliana* WASPs, there is less variability in signal shape found in literature, with our waveforms matching those previously reported. Still, when studying wound response in plants, some aspects must be considered. Firstly, surface recordings are more suitable for this end than intracellular approaches, since they are non-invasive and thus do not elicit wound responses caused by insertion. However, they do not allow for the identification of the cellular identity of these signals (Farmer et al., 2020). This leads to a very relevant conundrum in the study of plant wound response: not being able to identify cellular origins without invasive techniques; but also changing the wound response by using said techniques. This problem is now starting to be surmounted by the use of voltage-sensitive dyes and genetic mutants (Zhao et al., 2015; Farmer et al., 2020; Rigoulot et al., 2021). Nonetheless, a better integration of molecular-based approaches and electrophysiological techniques still needs to be developed.

Our study, albeit not tackling the issue of the cellular origin of plant electrical signals, was still able to show that, for *A. thaliana* WASPs, the OECT offers the advantage of a higher SNR when not in laboratory conditions compared to the used electrodes.

Overall, this study validates OECTs as viable tools for plant electrophysiology, with the clear advantage of maintaining signal integrity outside of laboratory conditions. The application of untethered OECT-based sensors *in situ* using Arduino devices with WiFi modules (Diacci et al., 2021) further corroborates this conclusion. Additionally, previous work on OECTs as mammalian electrophysiological tools employed multi-OECT arrays (Khodagholy et al., 2013), which implies that this technology can be used to resolve spatial and temporal signals in field conditions. A final advantage of OECTs is the possibility for their low-cost fabrication, using screen-printing technology (Zabihipour et al., 2020).

Alas, the validation of new tools for plant electrophysiology is only a small fraction of much needed innovation. Recent reviews cover the advances in plant-inspired biomimetic soft robotics and machines (Esser et al., 2020; Mazzolai et al., 2020), as well as the developments in plant biohybrid devices and bioelectronic applications (Dufil et al., 2021), highlighting the immense possibilities for interaction of plants with technology. However, without better and more diverse tools to study plant phenomena, these possibilities cannot be reached.

## Data availability statement

The raw data supporting the conclusions of this article will be made available by the authors, without undue reservation.

## Author contributions

AA-M designed and performed the experiments, analyzed the data, and wrote the manuscript. CD fabricated the OECTs, optimized the parameters for their use in these plant applications, and wrote the manuscript. AMD optimized the experimental setup and aided in the experimental design. MB and DTS contributed to the development of OECTs. ES conceived the project, designed, and supervised the research. All authors reviewed the manuscript.

## Conflict of interest

The authors declare that the research was conducted in the absence of any commercial or financial relationships that could be construed as a potential conflict of interest.

## Publisher’s note

All claims expressed in this article are solely those of the authors and do not necessarily represent those of their affiliated organizations, or those of the publisher, the editors and the reviewers. Any product that may be evaluated in this article, or claim that may be made by its manufacturer, is not guaranteed or endorsed by the publisher.
