# Supplementary material for: Benchmarking organic electrochemical transistors for plant electrophysiology
Source: Front Plant Sci. 2022 Jul 22;13:916120. doi: 10.3389/fpls.2022.916120 (PMC9355396; doi:10.3389/fpls.2022.916120)
Supplement: Supplementary file 1 [file Data_Sheet_1.docx]

Supplementary Material

Benchmarking organic electrochemical transistors for plant electrophysiology

Adam Armada-Moreira^1^, Chiara Diacci^1^, Abdul Manan Dar^1^, Magnus Berggren^1,2^, Daniel T. Simon^1^, Eleni Stavrinidou^1,2,3*^

# Supplementary Figures and Tables

## Supplementary Figures


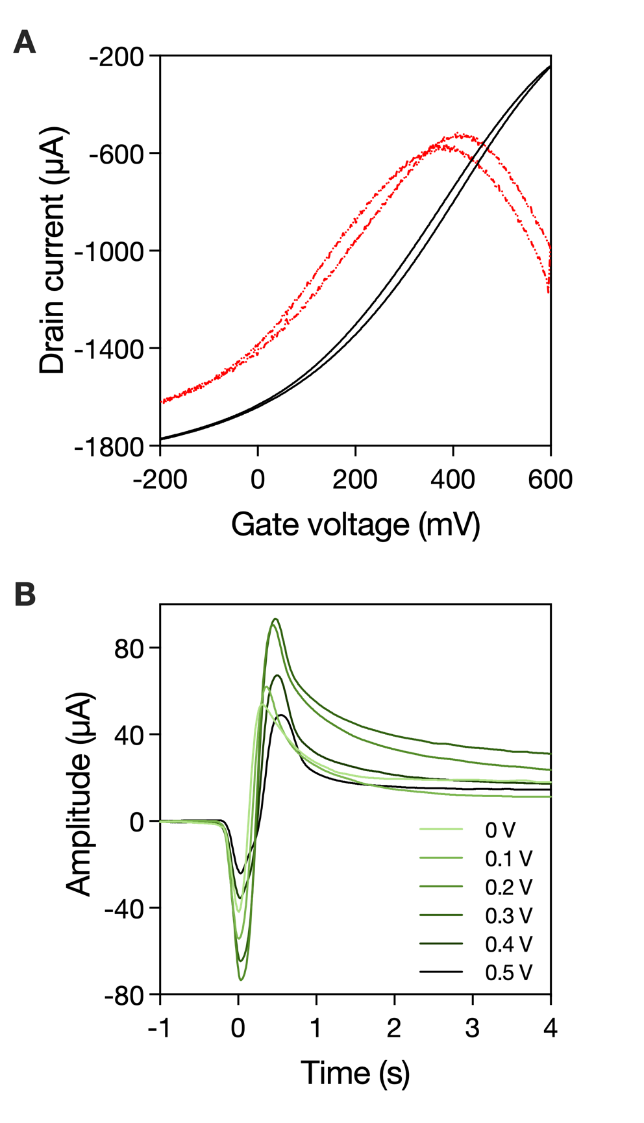


**Supplementary Figure 1.** Impact of different gate voltages in signal amplification. **(A)** Transfer curve of OECT with a source-drain bias of -0.4 V. Red trace represents the derivative of the transfer curve, showing the peak in transconductance at c. 0.3 V. **(B)** VFT action potentials recorded with different gate voltages and -0.4 V drain voltage.


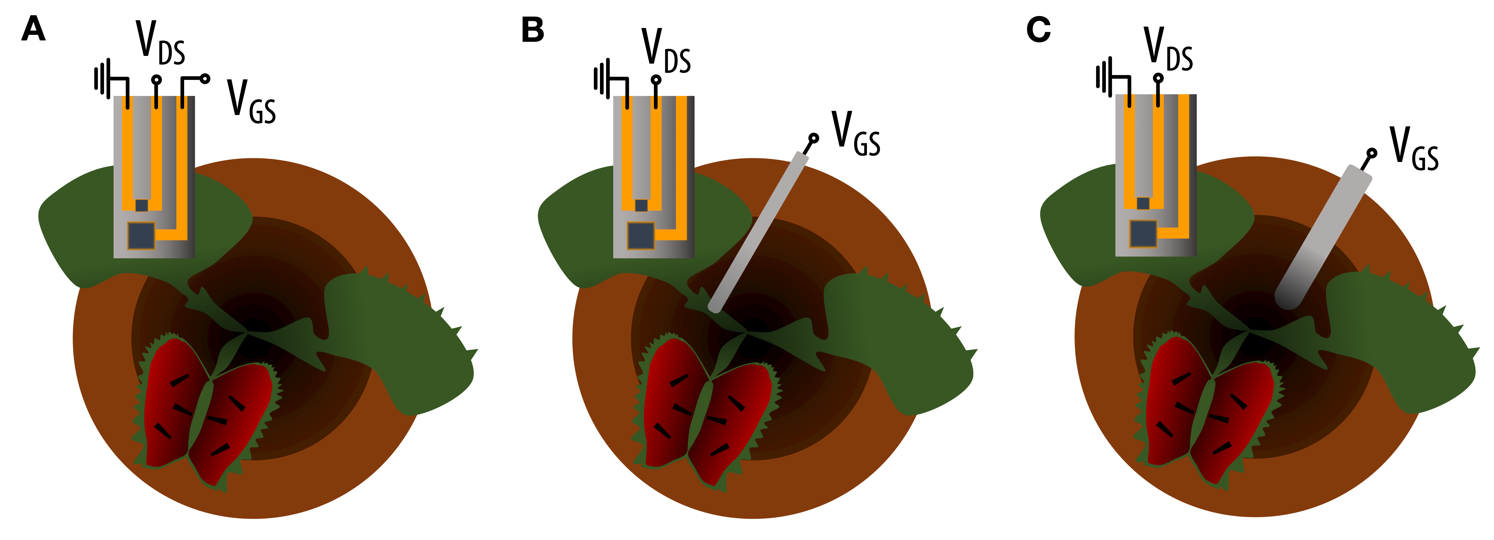


**Supplementary Figure 2.** Alternative circuit representation for the different OECT configurations for plant electrophysiology. **(A)** OECT co-planar PEDOT:PSS gate electrode. **(B)** Ag/AgCl electrode in a non-electrically active plant tissue as the gate electrode. **(C)** Ag/AgCl electrode in soil used as gate electrode.


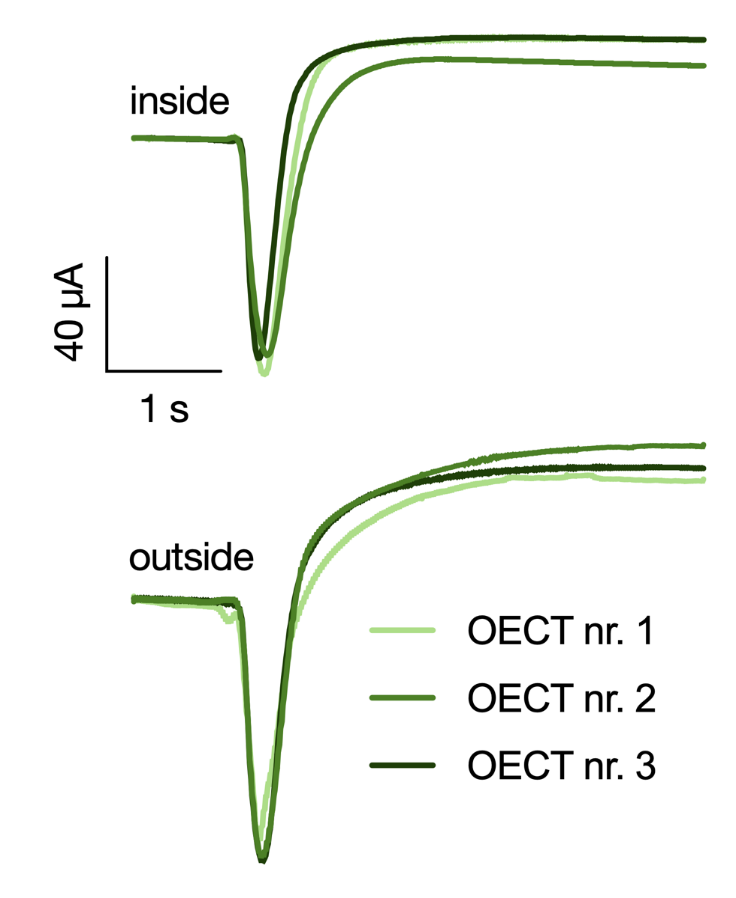


**Supplementary Figure 3.** Average VFT action potential recorded with different OECTs, inside and outside of the Faraday cage.


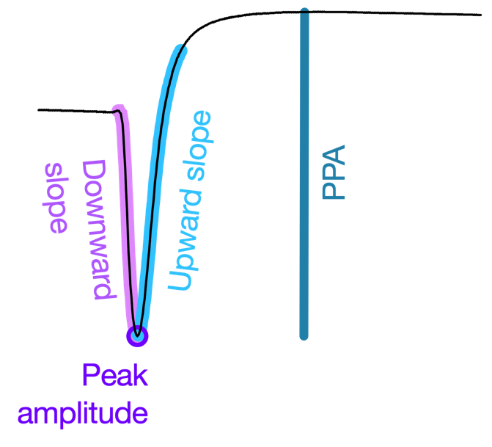


**Supplementary Figure 4.** Representation of the analyzed parameter relationship in the VFT action potential, where the linear relationships downward slope - peak amplitude and upward slope - peak-to-peak amplitude (PPA) were compared among the different devices.

## Supplementary Tables

**Supplementary Table 1.** Characterization of experimental datasets.

|  | **Inside Faraday cage** | | | **Outside Faraday cage** | | |
| --- | --- | --- | --- | --- | --- | --- |
|  | Devices | Plants | Traps/leaves | Devices | Plants | Traps/leaves |
|  | Venus flytrap | | | | | |
| OECT | 3 | 8 | 24 | 3 | 10 | 21 |
| Ag/AgCl | 1 | 4 | 9 | 1 | 6 | 18 |
| PEDOT:PSS | 1 | 6 | 14 | 1 | 5 | 13 |
|  | *Arabidopsis thaliana* | | | | | |
| OECT | 1 | 5 | 20 | 1 | 5 | 12 |
| Ag/AgCl | 1 | 4 | 15 | 1 | 3 | 12 |
| PEDOT:PSS | 1 | 4 | 10 | 1 | 4 | 13 |

**Supplementary Table 2.** Waveform correlation, represented as R^2^, between different devices in the same cage condition and same device in different cage condition.

|  | **Venus flytrap** | | ***Arabidopsis thaliana*** | |
| --- | --- | --- | --- | --- |
| *Similarity between different devices* | Inside | Outside | Inside | Outside |
| OECT-Ag/AgCl | 0.9536 | 0.8566 | 0.9952 | 0.9777 |
| OECT-PEDOT:PSS | 0.9104 | 0.9515 | 0.9224 | 0.8339 |
| Ag/AgCl-PEDOT:PSS | 0.9161 | 0.8433 | 0.9235 | 0.8510 |
| *Similarity of same device inside and outside* |  | |  | |
| OECT | 0.9850 | | 0.9948 | |
| Ag/AgCl | 0.9216 | | 0.9610 | |
| PEDOT:PSS | 0.9666 | | 0.9394 | |

**Supplementary Table 3.** Slope analysis of relationships between peak signal amplitude and slope, as well as PPA and slope between minimum and maximum amplitude for the Venus flytrap datasets. There were no significant differences between the slopes, using ANCOVA analysis of slope differences, leading to the conclusion that one curve fits all the datasets (p = 0.9442 and p = 0.6456 respectively).

|  |  | **Relationship between peak signal amplitude and slope** | | **Relationship between PPA and slope between minimum and maximum** | |
| --- | --- | --- | --- | --- | --- |
|  |  | Slope | R^2^ | Slope | R^2^ |
| OECT | inside | 0.0738 | 0.8222 | 0.0465 | 0.6054 |
|  | outside | 0.0787 | 0.8820 | 0.0513 | 0.7039 |
| Ag/AgCl | inside | 0.0860 | 0.9880 | 0.0531 | 0.7880 |
|  | outside | 0.0868 | 0.7349 | 0.0736 | 0.7367 |
| PEDOT:PSS | inside | 0.0828 | 0.7902 | 0.0573 | 0.5359 |
|  | outside | 0.0766 | 0.9404 | 0.0509 | 0.6694 |
| Combined |  | 0.0684 | 0.9128 | 0.0358 | 0.7292 |
